# Biophysical Stimulation in Delayed Fracture Healing of Hand Phalanx: A Radiographic Evaluation

**DOI:** 10.3390/biomedicines10102519

**Published:** 2022-10-09

**Authors:** Francesco De Francesco, Pasquale Gravina, Stefano Varagona, Stefania Setti, Antonio Gigante, Michele Riccio

**Affiliations:** 1 Department of Reconstructive Surgery and Hand Surgery, Azienda Ospedaliera Universitaria delle Marche, Via Conca, 71, 60126 Ancona, Italy; 2 Clinical Orthopedics, Polytechnic University of Marche, Via Conca, 71, 60126 Ancona, Italy; 3 IGEA SpA, Clinical Biophysics, Via Parmenide, 10/A, 41012 Carpi, Italy

**Keywords:** phalangeal fracture, non-union, delayed fracture, biophysical stimulation, electromagnetic field, PEMF, CCEF

## Abstract

Phalangeal fractures are common events among the upper limbs accounting for 10% of all human body fractures. Fracture complete healing process may persevere several months or years. Most phalangeal fractures present favorable union within 3 to 6 weeks. In the literature, biophysical stimulation has yielded favorable outcomes in the treatment of hand fractures. A survey involving hospitals in the US reported the use of biophysical stimulation (72%) in relation to nonhealing fractures at three months after trauma. A noninvasive procedure such as biophysical stimulation may be preferential prior to consideration of invasive procedures. In this retrospective study, we analyzed 80 phalangeal fractures, 43 of which did not show any radiographic sign of healing 30 days after surgery; on radiograms, we calculated radiographic data and the total active motion (TAM) for clinical comparison. All radiographic images were evaluated using Adobe Photoshop CS3 (version 10.0, Adobe Systems Inc., San Jose, CA, USA). We calculated the index of relative bone healing each month after surgery starting from 30 days, which was considered as T1, and followed up for a total of 6 months after stimulation (T6) with better results in stimulated groups. We concluded that prompt administration of biophysical stimulation supports fracture healing and yields an important improvement in the union rate compared with nontreatment. Above all, our patients experienced less injury-related distress between the fracture and repair period, which consequently reduced immobilization time, envisaging an early rehabilitation interval, with a better patient hand outcome.

## 1. Introduction

In Europe, the incidence of hand fractures in 2017 was evaluated to be 178.9 per 100,000 individuals cases and is expected to increase by 23% in 2030, with healthcare costs at EUR 37.5 million in 2017 estimated to rise by 27% in 2030 [1]. Hand fractures are more frequent in men than in women [2], and the most affected sites are the little, ring, and middle fingers rather than the thumb and index finger [3]. Phalangeal fractures are common events among the upper limbs accounting for 10% of all human body fractures [4] and 46% of all hand and wrist fractures, the most frequent after distal radius or ulnar fractures [5,6]. Such injuries mainly occur in young people and the elderly, causing a significant economic burden on the society. Phalanx fractures frequently are consequences of blunt trauma accidents, crush injuries, penetrating trauma, work-related incidents, car accidents, and sport injuries [7]. A small proportion of fractures, which are conservatively treated using specific casts, may indicate the presence of a tumor [8]. Complications have been observed in such fractures concerning stiffness and deformity due to early mobilization and have led to an increase in mandatory hand surgery. In some hand fractures, such has spiroid, articular, comminute, and open fractures, surgical treatment is always indicated, mainly in polytrauma with soft tissue lesions [9]. Fracture healing represents a complicated process occurring via direct or secondary recovery. For long bones, the complete healing process may persevere for several months or years. Bone is a unique connective tissue with a potential to entirely heal by cellular regeneration and mineral matrix production, resulting, to some extent, in a restitutio ad integrum as opposed to the simple deposition of collagen, which results in a scar [10]. Most phalangeal fractures present favorable union within 3 to 6 weeks [11,12]. Open fractures may require an extended healing time as reported by Smith and Rider based on radiographic signs of bone healing at 5 months following diagnosis of an open fracture [13]. Jupiter and colleagues considered failure in healing within a four–month period as a delayed union of a phalanx fracture [14]. A non-union may be delineated as failure in healing more than six months since the time of fracture, with no visible signs of progression to healing for three months [15,16]. In addition, treating physicians have also described a non-union as a fracture that is not able to heal without further therapeutic mediation [17]. The definition of pseudarthrosis derives from Greek meaning “pseudo-false” and “arthro-articulation”; that is, a nonconsolidated fracture forms a resemblance to a joint, characterized by a mobility of the nonhealed fragments, a “false” joint due to its atypical nature, lacking in ligaments and muscles. The percentage of delayed union has never been estimated, but a non-union represents 9% and a mal-union up to 28% of fractures [15]. Pseudarthrosis of long bones accounts for approximately 10% of fractures [18]. Several elements are involved in delayed or non-union fractures including the loss of bone substance, inadequate immobilization, sepsis, or systemic pathology such as diabetes [19]; smoking [20] may also be considered a risk factor, along with many other causes [21,22,23,24,25,26]. Before performing a new surgery, there are other weapons in our arms consisting in nonsurgical treatment, such as biophysical stimulation. In the literature, biophysical stimulation has yielded favorable outcomes in the treatment of hand fractures, specifically related to scaphoid fractures [27] and scaphoid non-union fractures [28]. Biophysical stimuli have been adopted during the last 50 years to enhance fracture healing [29]; preclinical research has revealed that biophysical stimuli interaction occurs on the cell membrane and, consequently, activates metabolic pathways within the cell. Cultured bone cells exposed to biophysical stimuli increase their proliferation and synthesis, as well as release growth factors pertaining to the family of TGF-β [30]. In animals, it has been shown that the stiffness of experimental osteotomies is substantially greater in treated animals compared with controls [31], and the mineralization rate of newly developed trabeculae is twofold higher [32]. The effect on osteogenesis depends on the physical parameters that characterize biophysical stimuli; those response curves have been repeatedly described. Three technologies are mainly used in clinical contexts, namely pulsed electromagnetic fields (PEMFs), low-intensity pulsed ultrasound (LIPUS), and capacitive coupling electric fields (CCEFs). PEMFs can be used with a cast, whereas the other technologies require application to the overlying skin of the fracture. In clinical settings, biophysical stimulation is used to accelerate fracture healing both in recent fractures and non-unions [33]. The presence of infection or a synthesis device is not contraindicated in the use of biophysical stimulation. Requirements for application include adequate immobilization and alignment of the fracture stumps; a potential bone gap will not exceed 50% of the bone diameter. Biophysical stimulation has been administered to heal non-unions with a success rate greater than 70% when promptly applied, and it is able to reduce healing time by 30% [34].

In the USA and Europe, the use of physical energy to enhance bone repair mechanisms has been investigated during the last century with approximately 10,000 patients undergoing treatment worldwide. A survey involving hospitals in the US reported the use of biophysical stimulation (72%) in relation to nonhealing fractures at three months after trauma [35]. A substantial proportion of Canadian orthopedic surgeons (45%) currently make use of bone stimulators as part of their clinical orthopedic management [36].

The aim of this study was to investigate bone regeneration through digital objective radiographic data in phalanx fractures with delayed union in the presence of biophysical stimulation.

## 2. Materials and Methods

### 2.1. Patients

This retrospective study was authorized by a local ethics committee (484/2021). All procedures involving human participants were performed according to the ethical regulations of the institutional and national research committee, conforming to the 1964 Declaration of Helsinki and later amendments or comparable ethical standards. Between January 2016 and December 2020, patients affected by delayed union in finger fractures were treated in our hospital.

Inclusion criteria involved patients presenting finger fractures associated with soft tissue lesions, who had undergone surgical fixation with delayed union at 30 days from surgery.

Exclusion criteria involved diabetic patients, heavy smokers, vasculopathy patients, infected wounds, nonsurgical fractures, and subjects presenting composed fractures with non-union gap < 1 mm.

Clinical data included (*i*) demographic characteristics and (*ii*) total active motion (TAM). The TAM considers the hand metacarpal phalangeal (MCP), proximal interphalangeal (PIP), and distal interphalangeal (DIP) according to the following formula: MCP + PIP + DIP of the affected digit minus the sum of the extensor lag of (MCP + PIP + DIP) of the same digit [37].

Radiographic data included (*i)* the fracture location of the metacarpal bone (M), proximal interphalangeal bone (F1), intermedium interphalangeal bone (F2), and distal interphalangeal bone (F3); (*ii*) type of fracture—diaphyseal (transverse, comminute, and spiroid) or articular (single fragment and comminute); and (*iii*) radiographic bone healing.

All clinical and radiological data were assessed at 30 (T1), 60 (T2), 90 (T3), and 180 days (T6).

### 2.2. Biophysical Treatment

The efficacy of PEMFs or CCEFs in bone healing has been widely documented in the international literature [33,34]. In our daily orthopedic experience, we offer the patient one of two methods, following this clinical indication: CCEF stimulation in patients with accessible skin areas or PEMF stimulation in the presence of non-healed wound or in presence of a splint or cast, since the CCEF requires direct skin contact. The medical device for CCEF stimulation (Osteobit^®^ IGEA Spa, Carpi, Italy) delivers a density current of 25 µA/cm^2^ in the relevant site. The standard electric signal developed contains electrical pulses of 12.5 Hz with a duty cycle of 50%. The electric pulse is part of an active process containing a sinusoidal wave of 60 kHz. The electrodes are equipped with a layer of highly conductive material coated with adhesive biocompatible conductive gel. Patient compliance in using Osteobit^®^ is very high because it is the only medical device for biophysical stimulation with the CCEF with adhesive electrodes completely dedicated to treating the phalanges: 1.5 cm × 5 cm. In patients bearing a splint or cast, PEMF stimulation (Biostim^®^ IGEA Spa, Carpi, Italy) was applied. The dedicated coil for hand was disposed on the operated finger and powered by the PEMF generator system, which delivers a pulsed signal containing a peak magnetic field intensity of 2.5 ± 0.1 mT and a frequency of 75 Hz. The patient was required to wear the battery-operated device (Biostim^®^ or Osteobit^®^) 8 h/day for 60 days during the daytime or night-time and to be alert to any undesirable events or symptoms including burning sensation or signs of skin rash, which would indicate immediate interruption of the treatment.

The device was to be worn for a minimal 8 h/day interval for 60 days. The patients who agreed to comply with the biophysical stimulation (PEMF or CCEF) regimen were included in the stimulated group and retrospectively compared with the control group of untreated patients who refused the postoperative biophysical application.

### 2.3. Radiological Evaluation

All radiographic images were evaluated using Adobe Photoshop CS3 (version 10.0, Adobe Systems Inc., San Jose, CA, USA) and were conducted with the same radiological device available in our health center. We evaluated the osseous defect site to analyze the radio density change over time with biophysical stimulation.

**Figure 1:**
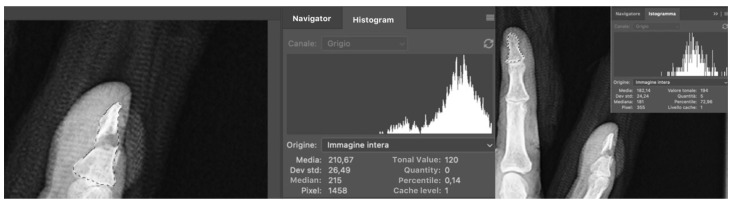
Histogram panel shows statistical results concerning ROI including mean value as average intensity value and standard deviation denoting differing intensity values; median displays range of intensity values; pixels indicate overall amount of pixels used to measure the histogram; total number of pixels corresponding to intensity level below the pointer; tonal value displays intensity level related to the area below the pointer; percentile shows aggregate amount of pixels at or below the pointer level indicating pixel percentage of the image, from 0% at extreme left to 100% at extreme right.

The conventional X-ray machine product images were in DCOM (Digital Imaging and Communications in Medicine) format and rendered by the program used in our hospital (PACSWEB) in JPEG (Joint Photographic Experts Group) digitized at 300 dpi so that all images underwent the same process to reduce the noise in tonal value research. The files were subsequently imported into Adobe Photoshop TM CS software. The region of interest regarding each image was selected using a standardized technique consisting of the selection of a region of interest (ROI) using the magnetic selector of Photoshop, which is able to distinguish the background from the bone analyzed with a precision of 1 pixel, to examine the optical density and ROI that consists of the shape of the analyzed bone cortical. A blinded assessor used a histogram plot to measure the mean density of the selected site in pixels (Figure 1).

The histogram panel shows the statistical results concerning the ROI including the mean value as the average intensity value and the standard deviation denoting the differing intensity values. Moreover, the median displays the range of intensity values; the pixels indicate the overall number of pixels used to measure the histogram; the total number of pixels corresponding to the intensity level below the pointer is also expressed. Tonal value displays the intensity level related to the area below the pointer; the percentile shows the aggregate number of pixels at or below the pointer level indicating the pixel percentage of the image, from 0% at the extreme left to 100% at the extreme right. We compared the total value of the defect area with the tonal value of the unaffected area [38,39]:

$$Index\;of\;relative\;bone\;healing\;\left(I\right)=\frac{Bone\;donsity\;of\;bone\;defect}{Bone\;density\;of\;surrounding\;bone}$$

Then, we statistically compared the index of relative bone healing before stimulation (T1) corresponding to the first X-ray exam performed at 30, 60 (T2), 90 (T3), and 180 days (T6) after surgery. The tonal value improves in parallel with the occurring healing process, and the higher the numeric value of the tonal value, the higher the percentage of calcium (no transparency at X-ray) and, in consequence, the higher the percentage of the bone in the analyzed segment. We statistically compared the tonal value before stimulation (T1) corresponding to the first X-ray exam performed at 30, 60 (T2), 90 (T3), and 180 days (T6) after surgery.

## 3. Statistical Analysis

Categorical variables are herein designated as absolute numbers, and the percentages were analyzed using contingency tables and the chi-square test for independence. Continuous variables and scores are reported as mean and standard deviation for each group and compared using Student’s t-test analysis; comparisons of repeated measurements within each group (T1 vs. follow-up values) were analyzed by a two-tail paired t-test corrected for multiple measurements; and comparisons between groups (at T1 and at each follow-up time) were conducted using a two-tailed heteroskedastic unpaired t-test. *p* values that were lower than 0.05 were considered as statistically significant. Statistical analyses were performed using NCSS 9 software (Hintze, J. (2013). NCSS 9. NCSS, LLC. Kaysville, UT, USA, www.ncss.com, accessed on 1 June 2022).

## 4. Results

**Figure 2:**
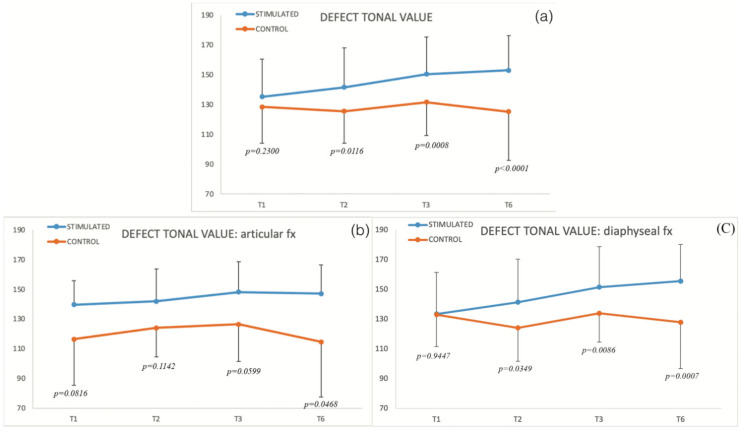
(**a**) Defect tonal value in stimulated and control groups; (**b**,**c**) show defect tonal value in subcategory articular and diaphyseal fractures (*p*, significant difference between groups).

Forty-three patients were included in the stimulation treatment group and were compared with the control group consisting of thirty-seven patients. The mean age of the patients in the two groups did not significantly differ (51.6 ± 13.4 years in the stimulated group vs. 53.3 ± 19.3 in the control group, *p* = 0.6414). The defect tonal value was calculated in each group at baseline (time of delayed fracture, T1) and reevaluated every month for the first 3 months and then at 6 months during the healing process (Figure 2). The defect tonal value (Table 1) non significantly increases compared with control in the stimulated group at T1 (*p* = 0.2300), significantly increases at T2 (*p* = 0.0116) and T3 (*p* = 0.0008), and remains significantly higher up to T6 (*p* < 0.0001).

If we compare the defect tonal value between stimulated and control group in articular and diaphyseal fractures, the first one is nonsignificant at T1 (*p* = 0.0816), T2 (*p* = 0.1142), T3 (*p* = 0.0599), and significant at T6 (*p* = 0.0468); and the second one is nonsignificant at T1 (*p* = 0.9447) and significant at T2 (*p* = 0.0349), T3 (*p* = 0.0086), up to T6 (*p* = 0.0007) (Figure 2b,c, Table 2).

Accordingly, we analyzed the relative bone density index during follow-up in the two groups. The results are reported in Table 3. The relative bone density index has been subcategorized for diaphyseal and articular fractures (Table 4).

**Figure 3:**
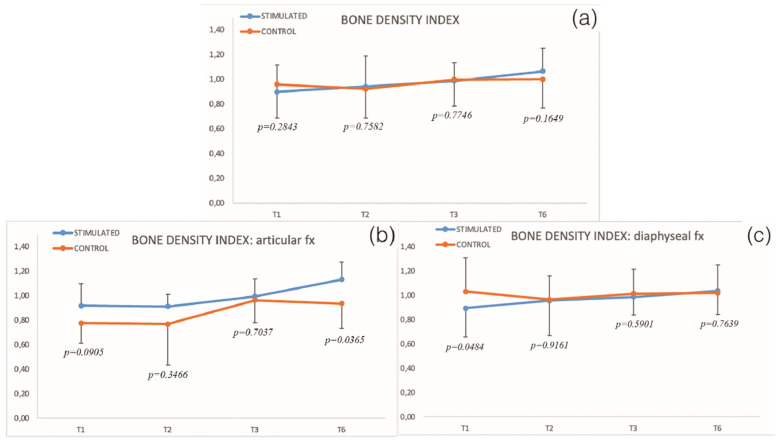
(**a**) Bone density index in stimulated and control groups; (**b**,**c**) bone density index in the subcategory articular and diaphyseal fractures (*p*, significant difference between groups).

The relative bone density index (Figure 3, Table 3) increases only in the stimulated group since T1 to T6, but a nonsignificant difference from T1 (*p* = 0.2843) to T6 is observed (*p* = 0.1649) between groups. 

No differences in relation to T1 are noted at follow-up for the control group. In stimulated group significant difference in relation to T1 was observed at T3 (*p* = 0.0431) and T6 (*p* = 0.0010) 

Comparing stimulated versus control group in diaphyseal and articular fractures, a statistically different result is at T1 (*p* = 0.0484) for the diaphyseal fracture and at T6 (*p* = 0.0365) for the articular fracture. 

**Figure 4:**
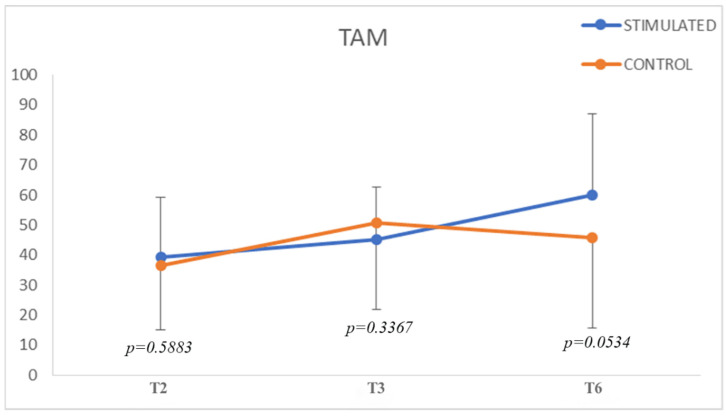
Total active motion (TAM) in stimulated and control groups.

**Figure 5:**
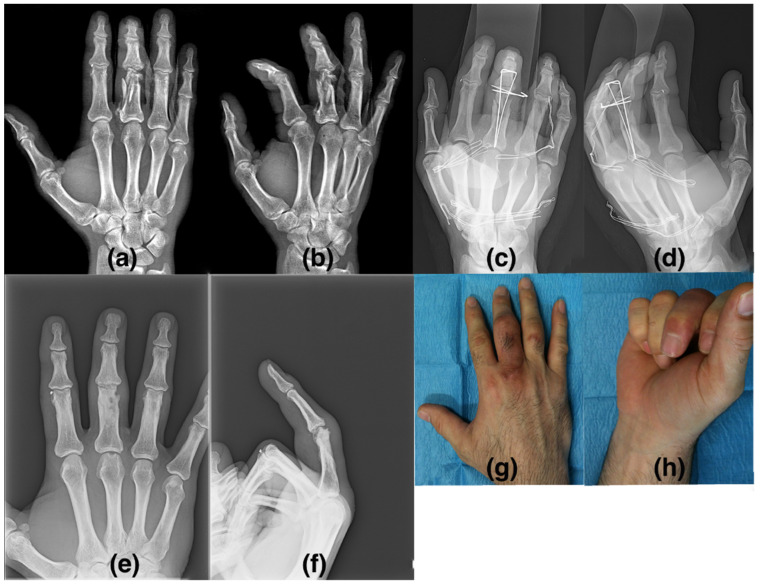
This figure shows clinical example of complex fracture treated in emergency theater that undergone biophysical stimulation. (**a**,**b**) show hand X-ray; we can notice the multiple fragmented fractures in anteroposterior view (**a**) and oblique view (**b**); (**c**,**d**) show X-ray image at T1, consisting of biophysical stimulation prescription; (**e**,**f**) show fracture healing at T6 in X-ray anteroposterior view (**e**) and later lateral view; (**g**,**h**) show good clinical result.

Data on TAM are reported in Table 5, where a significant increase in TAM is evident in both groups. Moreover, at T6 there is a marginal significant difference (*p* = 0.0534); in fact, based on our clinical evaluation, we can certainly point out that the stimulated group shows significant improvement over the control group in terms of joint ROM and, therefore, early recovery of hand function (Figure 4). A clinical example is shown in Figure 5.

## 5. Discussion

The hand is the most common skeletal site affected by fractures [40]. In Great Britain, Anakawe and colleagues, in 2011, conducted an epidemiological study of hand fractures resulting in 2.8 cases per 1000 people (prevalence with 62% and 38% of metacarpal and phalangeal fractures, respectively) [41]. In the USA, Karl and colleagues, in 2015, reported that finger fractures were extremely common events, representing 20% of all hand fractures, specifically phalangeal and metacarpal fractures at 0.125% and 0.084%, respectively [5]. Few publications have reported the incidence and prevalence data of hand fractures in Europe. De Jonge and colleagues conducted a study in Germany and reported that 0.2% to 3% of all patients presenting to the emergency department were affected by hand fractures [42,43]. Another study conducted in Norway showed an incidence of 29% of metacarpal fractures, 59% phalangeal, 14% V metacarpal, and 9% V F1 [44]. In Italy, 20.8% of hand traumas were open phalanx wounds, 7.9% phalanx fractures, and 4.1% metacarpal fractures [45]. The two major causes are traffic accident and machine-related injuries. An appropriate treatment fracture normally heals by bone alignment, and the complete healing is known as “bone union”; a “mal-union” occurs in the absence of an anatomical alignment in both bone segments or due to malrotation during the mispositioning of the segments. A “non-union” occurs in a nonhealing or inadequate healing of the fracture. The main causes of mal-union involve a fracture treatment delay, fracture instability, improper treatment, and patient noncompliance of conservative treatment. Furthermore, non- unions are observed in the fracture healing processes that fail in the local healing process of bone injury. Mills provided a satisfactory definition of a non-union as a fracture with no apparent sign of healing within three consecutive months from fracture [16]. Furthermore, a large investigation conducted by the abovementioned authors from 2005 to 2010 reported the rate of non-unions per fracture in almost 5.000 people including 238 hand fractures. The authors observed that the peak of non-union was between 35 and 44 years, 25–34 for men and 65–74 for women, and the overall percentage of hand fractures was 0.3% with a non-union rate of 1.5% for women and 2.3% for men. Noteworthy was the increase in the number of fractures as age advanced in contrast to the number of non-union fractures that remained constant, suggesting that osteoporosis is unrelated to non-union. The global risk of non-union seemed to be 1.9% with the highest risk in 25- to 44-year-old patients and the lowest risk in the elderly population [46]. Non-union represents an important therapeutic challenge ranging from an adequate or partially favorable outcome to permanent disability. A favorable outcome of hand fractures strictly depends on an appropriate rehabilitation and a gradual fracture healing process. The critical phases of healing are inflammation (first to second week), repair (second to sixth week), and remodeling (eighth week to one year). Passive range of motion and strengthening programs may not be initiated before the premature and final stages, respectively, which determine callus conversion to bone formation. An adequate splint may substitute the hard callus and indicates the onset of passive motion in the repair stage [47]. Phalangeal fractures respond to immobilization and predict the return of motion at approximately 84%; metacarpal fractures provide a more favorable outcome of 96% [48]. However, the percentage will drop to 66% in the event of the immobilization period exceeding four weeks. The aforementioned findings imply the essential role of rehabilitation in hand fracture outcomes and the urgency of early administration [49]. A diagnosis of delayed union is generally based on both clinical and radiological findings. Radiography has always been the most used technique to assess fracture healing, and the most studied parameters are the external callus and bridging of the fracture line by a callus [50]. Several scores, specific or not, have been created to assess fracture healing, and the most reliable method was introduced by Whelan and colleagues in 2002 [51]. Following this method, more specific methods for tibial [52], hip and femur [53,54,55], and radius [56] fractures were described. Additionally, X-ray fracture detection is one of the most common trigger points in trauma patients in multiple clinical settings, and clinical misinterpretations of fracture healing can represent damaging diagnostic errors. For this reason, an objective evaluation of radiographic assistance, through the analysis of the defect tonal value and bone density index, improves sensitivity and can even improve the specificity of fracture detection by radiologists and nonradiologists. Biophysical stimulation is an evidence-based treatment, as documented by Yuan and colleagues (2018), who reported the safety of biophysical stimulation. Moreover, the authors proposed the stimulation technique as a forthcoming, noninvasive treatment to enhance bone regeneration, which has contributed to further research in the past few years [57]. In this retrospective study, the clinical application of biophysical stimulation was assessed in postoperative delayed union of phalangeal fractures for the first time. We analyzed two groups of patients affected by hand fractures treated with surgery and biophysical stimulation and without stimulation. In terms of the defect tonal value, we reported a significant difference with respect to baseline at each follow-up only in the stimulated group. A significant difference was reported between stimulated and control groups at each follow-up, also. This result highlights how biophysical stimulation can improve calcium deposition, such as to progressively change the ROI intensity (defect tonal value) in the stimulated group during follow-up. This result evidenced a significative improvement of fracture healing in terms of radiographic quality and bone repairing when compared with the control group. Moreover, the relative bone density index increased only in the stimulated group with a significant difference with respect to baseline at T3 and T6 but not between groups. This result shows that bone repairing between groups is similar such that both reach a good radiographic healing at T3. Otherwise, if we analyze the fracture according to type (articular vs. diaphyseal), we can observe that, for articular fracture, the stimulated group reached a bone consolidation at T6 in a more significative way with respect to the control group (*p* = 0.0365), demonstrating how biophysical stimulation is more efficient, in particular, in fractures of the diaphyseal type, offering an earlier mobilization for the patient with an improvement of ROM basically significant (*p* = 0.0534) with respect to the nontreated group at T6. The total active motion thus yielded significant improvement in both groups; however, we suggest an early application of biophysical stimulation in delayed union to prevent non-union. Benefits of early application were observed in patients in terms of fracture healing and decreased immobilization. Following six months after trauma, patients who were treated via stimulation showed a percentage of healing of 86% among all fractures, calculated as the percentage of all the analyzed fractures that undergone healing in the active group, considering as not healed the ones who had pseudarthrosis. Among the multiple approaches explored to treat delayed union and non-union fractures, the majority of studies considered the use of invasive procedures, such as surgical debridement, bone grafting, and harvesting [58]. For these reasons, a noninvasive method, such as biophysical stimulation, may be preferred prior to consideration of invasive procedures [59]. In the presence of non-union, different authors suggested treatment with biophysical stimulation for bone healing [60,61,62]. In these trials, the authors presented the overall success rate of the patients without clarifying the impact of an early application of biophysical stimulation and its involvement in manual activities. Moreover, on the comparison of fracture types, specifically articular and diaphyseal fractures in both groups, significant improvement was observed in the stimulated group in relation to the control group. A relevant outcome was reported in the defect tonal value both for the articular and diaphyseal stimulated groups. Our study revealed the favorable effects of biophysical stimulation in fractures with multiple fragments, loss of bone substance, comminution, vascular impairment, open wounds, and all other non-union risk factors. We suggest prompt administration of biophysical stimulation following surgery in this kind of hand fracture with predictable delayed union to promote rapid bone healing and reduce immobilization time. Additionally, an early rehabilitation program is recommended to improve outcomes. Cost-effectiveness and the cost–benefit ratio of biophysical stimulation for fresh phalangeal fractures of the hand are key factors to consider. In this context, the authors argue that the expense for the patient is inversely proportional to the speed of recovery with regard to early return to work compared with nontreatment patients. For this reason, the National Institute for Insurance against Accidents at Work in Italy endorses the use of this medical device to ensure prompt resumption of work and social activities. However, it is noteworthy to consider related factors that may be attributable to the outcome, such as the difficulty in establishing accident-related local injury, and to standardize patient activity and specifically weight-bearing during the study period. Moreover, in case of complex trauma with comminute fractures, local as well as surgical variability is another accountable element. Another drawback of the present study was the comparatively small population sample for the fracture sites and the different fixation procedures adopted. On the other hand, the main strengths of this study are the use of biophysical stimulation in complex fractures of the phalanges and early application of the treatment, after 30 days from the unconsolidated fracture. Moreover, the lengthy duration of biophysical stimulation was related to a trend toward an increased union in the course of time. A limitation of the study is the absence of multivariate analysis.

## 6. Conclusions

In conclusion, prompt administration of biophysical stimulation supports fracture healing and yields an important improvement in the union rate compared with nontreatment. Above all, our patients experienced less injury-related distress between the fracture and repair period, which consequently reduced immobilization time, envisaging an early rehabilitation interval. From a biological perspective, benefits are evident in the recruitment of human-bone-marrow stromal cells, acceleration of osteoblast proliferation and differentiation, enhanced mineralization process, inhibition of osteoclast differentiation with protective effect against osteolysis, and stimulation in the production of bone matrix induced by growth factors.

## Figures and Tables

**Table 1:** Defect tonal value between stimulated and control group (*p* Value in bold means significant difference).

Defect Tonal Value									
	Stimulated				Control				*p* Value between Groups
	Mean	St.Dev	N	*p* value vs. T1	Mean	St.Dev.	N	*p* value vs. T1	
**T1**	135.2	25.2	43		128.5	24.3	37		0.2300
**T2**	141.6	26.6	33	**0.0609**	125.5	21.3	27	0.5495	**0.0116**
**T3**	150.5	25.0	41	**0.0033**	131.6	22.3	35	0.6200	**0.0008**
**T6**	153.1	23.3	43	**0.0002**	125.2	32.5	36	0.6962	**0.0001**

**Table 2:** Defect tonal value between stimulated and control groups subcategorized in articular and diaphyseal fractures (*p* Value in bold means significant difference).

Defect Tonal Value														
	*Articular Fractures (ART)*						*Diaphyseal Fractures (DIA)*							
	Stimulated			Control			Stimulated			Control			ART	DIA
	*Mean*	*St.Dev*	*N*	*Mean*	*St.Dev*	*N*	*Mean*	*St.Dev*	*N*	*Mean*	*St.Dev.*	*N*	Stimulated vs. Control	Stimulated vs. Control
**T1**	139.6	16.3	12	116.4	31	8	133.5	28.0	29	133.1	21.5	27	0.0816	0.9447
**T2**	142	21.6	10	124.0	19.6	6	141.5	28.9	23	124.2	22.	19	0.1142	**0.0349**
**T3**	148.2	20.4	13	126.5	25.1	8	151.6	27.1	28	134.0	19.4	25	0.0599	**0.0086**
**T6**	147.1	19.4	13	114.7	37.1	8	155.7	24.6	30	128.0	31.2	26	**0.0468**	**0.0007**

**Table 3:** Bone density index in stimulated and control groups (*p* Value in bold means significant difference).

Relative Bone Density Index									
	Stimulated				Control				*p* Value between Groups
	Mean	St.Dev	N	*p* Value vs. T1	Mean	St.Dev.	N	*p* Value vs. T1	
**T1**	0.90	0.22	43		0.96	0.27	37		0.2843
**T2**	0.94	0.25	33	0.5305	0.92	0.24	27	0.5197	0.7582
**T3**	0.99	0.15	41	**0.0431**	1.00	0.21	34	0.5524	0.7746
**T6**	1.06	0.19	43	**0.0010**	1.00	0.23	35	0.4957	0.1649

**Table 4:** Bone density index in stimulated and control groups subcategorized in articular and diaphyseal fractures (*p* Value in bold means significant difference).

Relative Bone Density Index														
	*Articular Fractures (ART)*						*Diaphyseal Fractures (DIA)*							
	Stimulated			Control			Stimulated			Control			ART	DIA
	*Mean*	*St.Dev.*	*N*	*Mean*	*St.Dev.*	*N*	*Mean*	*St.Dev.*	*N*	*Mean*	*St.Dev.*	*N*	Stimulated vs. Control	Stimulated vs. Control
**T1**	0.92	0.18	12	0.78	0.17	8	0.89	0.23	31	1.03	0.28	27	0.0905	**0.0484**
**T2**	0.91	0.10	10	0.77	0.33	6	0.96	0.29	23	0.96	0.19	19	0.3466	0.9161
**T3**	0.99	0.14	13	0.96	0.18	8	0.99	0.15	28	1.01	0.20	24	0.7037	0.5901
**T6**	1.13	0.14	13	0.94	0.20	8	1.04	0.20	30	1.02	0.23	25	**0.0365**	0.7639

**Table 5:** Total active motion between stimulated and control groups (*p* Value in bold means significant difference).

Total Active Motion (TAM)									
	Stimulated				Control				*p* Value between Groups
	Mean	St.Dev	N	*p* Value vs. TAM T2	Mean	St.Dev.	N	*p* Value vs. TAM T2	
**TAM T2**	39.2	20.1	37		36.5	21.6	35		0.5883
**TAM T3**	45.1	17.7	35	**0.0003**	50.9	28.9	31	**0.0003**	0.3367
**TAM T6**	60.1	27.0	32	**0.0000**	45.7	30.0	15	**0.0178**	0.0534

## Data Availability

The datasets generated during and/or analyzed during the current study are available from the corresponding author upon reasonable request.
